# Importance of respect for the menstrual cycle in sports training and competition: a nationwide multisport coach questionnaire

**DOI:** 10.3389/fspor.2026.1906962

**Published:** 2026-09-03

**Authors:** Christian Tinner, Helen Moser, Magdalena Jetschin, Jan Gewiess, Wolf Hautz, Sebastian Wangler

**Affiliations:** 1 Department of Orthopaedic Surgery and Traumatology, Inselspital, Bern University Hospital, University of Bern, Bern, Switzerland; 2 Department of Emergency Medicine, Inselspital, Bern University Hospital, University of Bern, Bern, Switzerland

**Keywords:** athlete health, coach education, female athletes, injury risk, menstrual cycle, sports coaching, women in sports

## Abstract

**Background:**

Emerging evidence suggests that menstrual cycle phase may influence injury risk in female athletes, yet whether coaches possess the awareness and tools to act on this information remains unknown in the Swiss sporting context.

**Methods:**

A nationwide, cross-sectional questionnaire-based study was conducted among active coaches working with female athletes across multiple sports and competitive levels in Switzerland. Data were collected electronically between October and December 2025. Outcomes included self-rated knowledge, attitudes toward cycle-based training, current implementation practices, perceived barriers, and hypothetical injury risk decision-making.

**Results:**

A total of 261 coaches completed the survey (51% female, 49% male). Self-rated general knowledge of the menstrual cycle was moderate (mean 6.4/10), while self-rated domain-specific knowledge related to performance, recovery, and injury risk was lower (mean 5.8/10). Female coaches reported significantly higher self-rated general knowledge than male coaches (*p* < 0.0001), whereas self-rated domain-specific knowledge did not differ significantly between genders (*p* = 0.168). Despite this, attitudes toward cycle-based training were strongly positive across all genders, with coaches rating perceived usefulness at 7.4/10 and willingness to learn at 8.5/10, with no significant gender difference. Implementation, however, was limited: 45% of coaches did not track the menstrual cycle at all, and only 10% actively considered it in training planning, with no significant gender differences in practice. Only 34 of 261 coaches (13%) reported observing specific injuries occurring more frequently in particular cycle phases. When presented with hypothetical risk scenarios, 172 of 200 coaches (86%) indicated they would adapt training, and 138 of 198 (70%) would consider recommending competition avoidance, at some level of elevated hypothetical injury risk. Gender differences emerged in competition-related decision-making, with female coaches showing greater tendency to maintain competition participation (*p* = 0.003). The most frequently reported barriers were lack of knowledge (68%), insufficient coverage in coaching education (53%), and lack of practical tools (36%).

**Conclusion:**

Swiss coaches recognize the relevance of the menstrual cycle for female athlete health and demonstrate strong willingness to adapt practices when supported by evidence. However, a substantial gap between awareness and implementation persists, driven by insufficient education and the absence of institutional guidelines. Targeted educational interventions and evidence-based injury risk data are needed to translate coach receptiveness into practice.

## Introduction

The rapid rise of female professional sports has been accompanied by increased competition and a greater emphasis on gender-specific research. Emerging evidence indicates that hormonal fluctuations during the menstrual cycle can affect physiological and psychological factors related to performance, including metabolism, thermoregulation, mood, and vulnerability to injury (1).

While some studies report modest phase-dependent variations—such as slightly improved aerobic performance or strength during the follicular phase and increased fatigue or decreased performance during the mid-luteal phase—the overall evidence remains inconsistent (2–5). Research involving endurance-trained athletes often shows no significant effects of menstrual cycle phase on key performance outcomes at the group level, despite significant inter-individual variability (6). Similarly, although follicular phase–based resistance training may offer small benefits in strength or hypertrophy for some athletes, most findings indicate that anaerobic performance and strength are mostly unaffected by menstrual cycle phase (7). Importantly, subjective symptoms and individual hormonal responses seem to have a greater impact on perceived performance than on objective measures (8, 9).

Beyond performance, menstrual cycle characteristics may also be linked to injury risk. Some studies indicate that menstrual irregularities are associated with a higher occurrence of musculoskeletal injuries (10). At the same time, hormonal contraceptive use might influence injury rates, possibly offering a protective effect, although the evidence remains inconclusive (11, 12). Additionally, the phase of the menstrual cycle has been connected to concussion susceptibility and recovery, though the evidence is limited (13, 14).

Most recent research on injury risk mainly targets knee injuries. Female athletes, particularly in football (soccer), face a notably higher risk of anterior cruciate ligament (ACL) injuries, with incidence rates reported to be 2–8 times greater than those in male athletes (15–17). Some studies indicate that injury susceptibility might rise during the ovulatory phase; however, detailed data on overall injury rates across various sports and menstrual cycle phases are still scarce (18).

Therefore, while cycle-informed and personalized approaches may benefit some athletes, current evidence does not support making universal changes to training or competition schedules solely based on menstrual cycle phase (9). In contrast, the potential link between menstrual cycle phase and injury risk—particularly ACL injuries—represents a more actionable domain. Established neuromuscular training programs already reduce ACL injury risk in female athletes by up to 61% (19).

For cycle-informed injury prevention to be feasible in practice, however, a fundamental prerequisite must be met: coaches need to be aware of the issue, and athletes must actively track their cycles. Existing evidence from other countries suggests that menstrual health literacy is low across the sporting community. In large-scale surveys, the majority of athletes rated their coaches’ knowledge of menstrual cycle effects as poor or very poor, and fewer than one in eight athletes reported discussing menstrual health with their coach. Male coaches in particular report feeling ill-equipped to address how the menstrual cycle affects performance, injury risk, and recovery (20). Whether comparable knowledge gaps exist within the Swiss sporting landscape remains unknown.

The aim of this study is to assess coaches’ self-rated knowledge of and attitudes toward menstrual cycle effects on training performance and injury risk, their current cycle tracking and implementation practices, and the perceived need for evidence-based guidance, across different sports and competitive levels in Switzerland. Throughout this manuscript, cycle-based training refers to the deliberate structuring of training content, load, or timing in relation to a female athlete's menstrual cycle phase — for example, scheduling higher-intensity sessions in a particular phase, or reducing volume during menstruation. It does not refer to the mere recording of cycle data, which we describe separately as cycle tracking.

## Methods

### Study design

A cross-sectional, questionnaire-based study with nationwide recruitment was conducted to assess coaches’ perceptions, knowledge, and practices regarding the importance of the menstrual cycle in relation to training performance and injury risk in female athletes. The study included coaches from multiple sports and competitive levels across Switzerland.

### Participants and recruitment

Eligible participants were active coaches working with female athletes in organized sports at recreational, competitive, and national elite levels. Coaches from a wide range of sports disciplines (including team and individual sports) were invited to participate. Recruitment was conducted with the support of Swiss Olympic, which provided a list of all affiliated national sport federations in Switzerland. The questionnaire was distributed via email to all listed federations, which were asked to forward the invitation to their coaches working with female athletes. All responses received during the study period were included. No reminder e-mails were sent. Participation was voluntary and anonymous. Coaches were categorized by employer and performance level. Coaches of local sports clubs were classified as club-level, while those coaching higher regional squads were categorized as regional-level. Coaches working in the highest leagues and employed by national sports associations were grouped at the national level.

### Questionnaire development

A structured questionnaire was purpose-built for this study in July 2025. At that time, no validated instrument assessing coaches’ knowledge and practices regarding the menstrual cycle was available to us. Items were drafted by the investigators and reviewed for content and comprehensibility by specialists in sports medicine, sports science, and gynaecological endocrinology, and refined accordingly. No pilot study was conducted and no formal psychometric validation was performed. The questionnaire was designed to evaluate:

- Perceived importance of the menstrual cycle in training and competition
- Coaches’ knowledge regarding menstrual cycle–related effects on performance and injury risk
- Current coaching practices and adaptations related to the menstrual cycle
- Perceived need for education and evidence-based guidelines

The questionnaire was organized into 7 sections: (1) demographic information, (2) knowledge and opinion, (3) cycle tracking/tracking tools, (4) reporting of cycle-related injuries, (5) implementation of cycle-based training, (6) barriers/lack of knowledge (7) free comments. It included multiple-choice questions, Likert scale items (10-point Likert scale), and open-ended questions to gather both quantitative and qualitative data (Supplementary Table 1). Prior to use, the questionnaire was reviewed for content and comprehensibility by the involved experts in sports medicine, sports science, and gynecological endocrinology and refined accordingly; no formal pilot study was conducted. A 10-point Likert scale was chosen for the knowledge, opinion, and willingness items to allow finer differentiation of responses than a conventional 5-point scale. For the knowledge items, the scale was anchored at 1 (“no knowledge”) and 10 (“expert”); for opinion and willingness items, higher values indicated greater perceived usefulness or willingness, respectively.

### Data collection

The questionnaire was administered electronically through an online survey platform (Microsoft Forms) between October and December 2025. Data were collected over a two-month study period. To ensure anonymity, no personal identifying information was gathered. Participants could complete the questionnaire only once.

### Outcome measures

As no study protocol was prepared in advance, outcomes were not formally pre-specified. The questionnaire assessed four domains:

- Self-rated knowledge: two 10-point items covering general knowledge of the menstrual cycle and knowledge of its effects on performance, recovery, and injury risk
- Attitudes: two 10-point items covering the perceived usefulness of cycle-based training and willingness to learn more
- Current practice: categorical items on cycle tracking, systematic recording, and consideration of the menstrual cycle in training planning

The fourth domain was hypothetical decision-making, assessed through two scenario items on training modification and on competition withdrawal at varying levels of elevated injury risk, together with items on observed phase-specific injuries, the use of prevention programmes, the existence of organisational guidelines, and perceived barriers to implementation.

### Statistical analysis

Statistical analyses were performed using R version 4.5.2 (R Core Team, 2025). Continuous data are presented as mean (95% CI) and categorical data as counts and percentages, with *P* < 0.05 considered statistically significant. N refers to the number of participating coaches in each group. Data distribution was checked with the Shapiro–Wilk test. When the normality assumption was met, continuous variables were compared between the two independent groups (male vs. female coaches) using an independent-samples Student's t-test; otherwise, the Mann–Whitney U test (Wilcoxon rank-sum test) was used. Categorical variables were compared between groups using the Chi-square or Fisher's exact test, as appropriate; for variables with more than two categories, the Fisher-Freeman-Halton exact test was used. Effect sizes are reported for all group comparisons: Cramér's V for categorical variables with more than two categories, the phi coefficient for 2 × 2 tables, and the rank-biserial correlation for Mann–Whitney U comparisons. Exact *p* values for contingency tables larger than 2 × 2 were obtained by Monte Carlo simulation. Where the omnibus test for a categorical variable with more than two categories was significant, *post-hoc* comparisons were performed for each category by collapsing the table into a 2 × 2 comparison (the respective category vs. all others, by group), using the Chi-square or Fisher's exact test depending on the expected cell counts, with Holm correction for multiple comparisons. No *post-hoc* comparisons were performed where the omnibus test was not significant; any category-level values presented elsewhere are descriptive and exploratory and were not tested. Not all coaches answered every item; each analysis was therefore based on the coaches who provided a valid response to the respective question (complete-case analysis), and no imputation of missing values was performed. The number of respondents contributing to each analysis is reported in the corresponding figures and tables.

### Ethical considerations

The study was conducted in accordance with the Declaration of Helsinki. Participation was voluntary, and informed consent was obtained electronically prior to completing the questionnaire. The project was submitted to the Cantonal Ethics Committee Bern, which determined that it does not fall within the scope of the Swiss Human Research Act (HRA) and therefore requires no authorisation by an ethics committee (declaration of non-jurisdiction, BASEC Req-2026-00625). The survey was anonymous and collected no health-related personal data on identifiable persons, neither on the responding coaches nor on their athletes; data protection requirements were observed throughout. No study protocol was prepared in advance, and the study was not registered in a trial or study registry. Given the descriptive, exploratory nature of a cross-sectional survey with no intervention and no clinical endpoints, prospective registration is not customary in this field and was not undertaken. We acknowledge that pre-registration of the outcome definitions and analysis plan would have strengthened the transparency of the present work.

## Results

### Demographic information

A total of 261 coaches completed the survey, of whom 51% were female and 49% male. As the invitation was forwarded by the federations, the number of coaches reached is unknown and a response proportion cannot be reported. Most respondents coached at club level (61%), followed by national (19%) and regional level (18%). Coaches represented a broad range of sports, with team ball sports being the most prevalent, followed by water sports and winter sports (Table 1).

**Table 1:** Distribution of coaches across different sports and performance levels. Percentages are calculated based on the total number of coaches within each sport. Coaches may be active at multiple levels; therefore, counts across levels may exceed the total number of coaches per sport.

Sport	Total (n)	Club level	Regional	National	Self-employed
Track and field	47	38 (80.9)	17 (36.2)	10 (21.3)	2 (4.3)
ball sports (handball, soccer)	127	108 (85)	29 (22.8)	16 (12.6)	0
water sports (rowing, swimming)	45	35 (77.8)	7 (15.6)	10 (22.2)	3 (6.7)
winter sports (skiing/snowboarding/bobsleigh)	21	2 (9.5)	4 (19)	20 (95.2)	1 (4.8)
endurance sports/running	11	6 (54.4)	7 (63.6)	4 (36.4)	3 (27.3)
other	19	12 (63.2)	2 (10.5)	8 (42.1)	3 (15.8)

**Figure 1:**
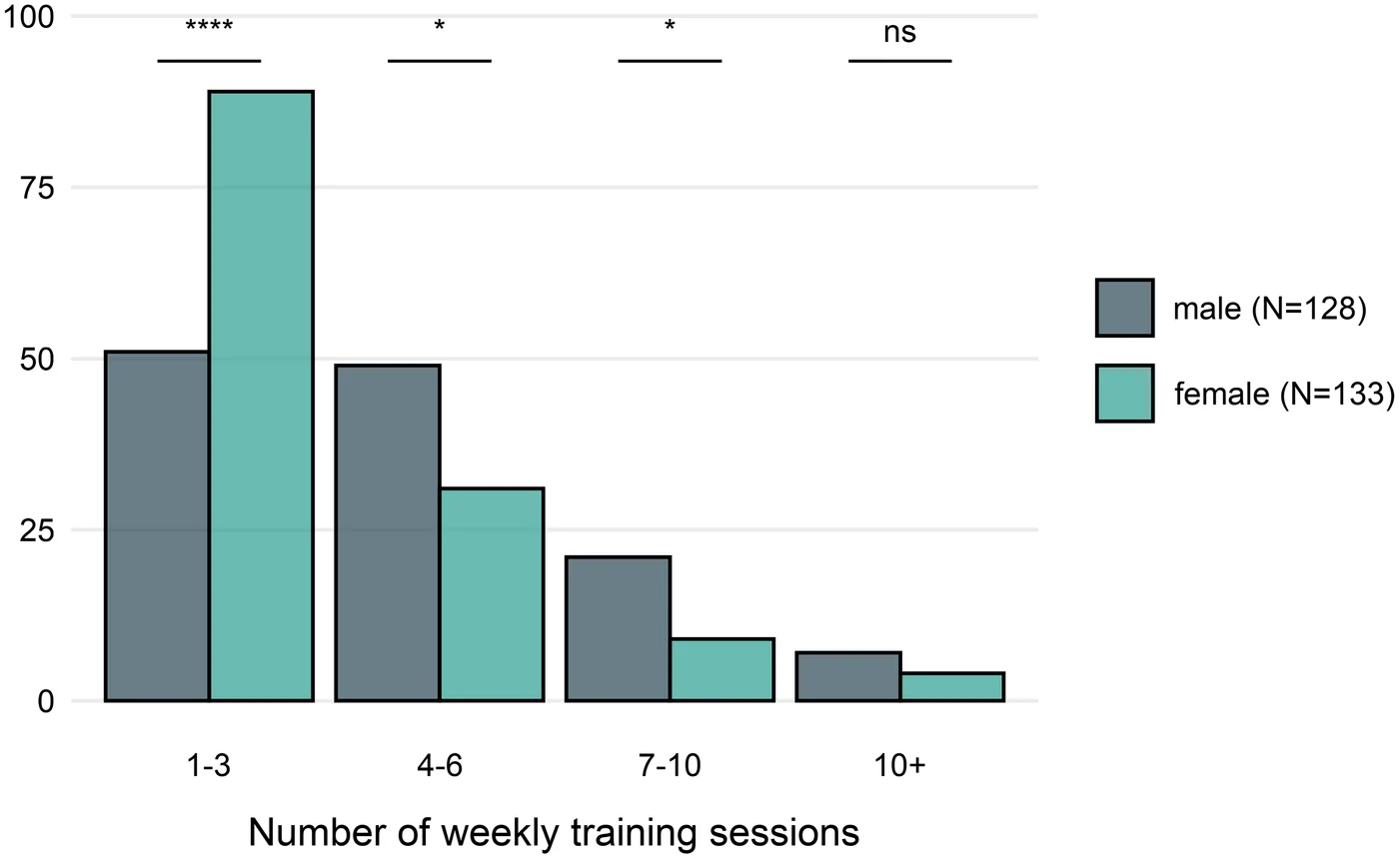
Bar chart comparing the number of weekly training sessions between male (*N* = 128) and female (*N* = 133) coaches. Overall difference between groups: Fisher-Freeman-Halton exact test, *p* = <.001, Cramér's V = 0.28. Significant differences were observed for: 1−3 (male 39.8%, female 66.9%, adj. *p* = <.001); 4−6 (male 38.3%, female 23.3%, adj. *p* = 0.038); 7−10 (male 16.4%, female 6.8%, adj. *p* = 0.049). No significant differences for: 10+ (male 5.5%, female 3.0%, adj. *p* = 0.370). Post-hoc: Chi-square/Fisher's exact tests, Holm-corrected.

Female coaches were significantly more likely to lead one to three practices per week, whereas male coaches more frequently led four to ten practices per week (Figure 1). No significant gender differences were observed among coaches leading more than ten practices per week. Additionally, a significantly higher proportion of female coaches worked in voluntary roles, while slightly more male coaches were employed in part-time or full-time roles.

### Knowledge and opinion

Self-rated general knowledge of the menstrual cycle was moderate (mean 6.4/10, 1 = no knowledge, 10 = expert), while self-rated knowledge specifically related to performance, recovery, and injury risk was lower (mean 5.8/10).

**Figure 2:**
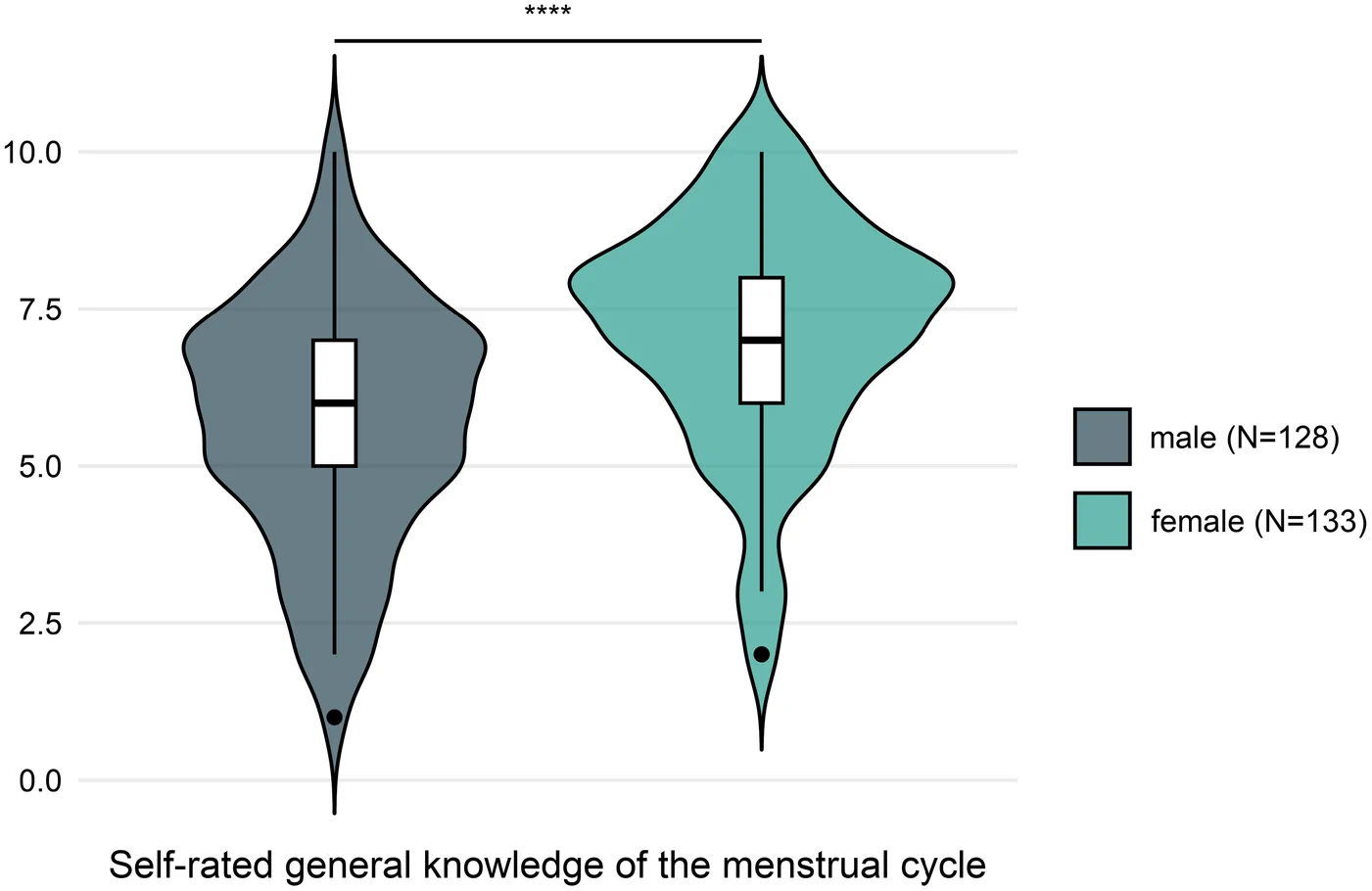
Violin plot comparing self-rated general knowledge of the menstrual cycle between male (*N* = 128) and female (*N* = 133) coaches (1 = very little, 10 = very extensive). Male: median 6.0 (IQR 5.0–7.0); female: median 7.0 (IQR 6.0–8.0). A significant difference was indicated (Mann–Whitney U, *p* < 0.0001; rank-biserial r = 0.44, 95% CI 0.31 to 0.54).

Female coaches reported significantly higher self-rated general knowledge than male coaches (Figure 2). For self-rated domain-specific knowledge relating to performance, recovery, and injury risk, no significant gender difference was found (male median 6.0, IQR 4.0–7.0, *n* = 128; female median 6.0, IQR 4.0–8.0, *n* = 133; Mann–Whitney U, *p* = 0.168). Notably, 33 of 261 respondents (13%) reported having received no prior information on the menstrual cycle in a sports context (male 14 of 128, 11%; female 19 of 133, 14%). This difference was small and was not formally tested.

### Interest in learning and attitudes toward cycle-based training

**Figure 3:**
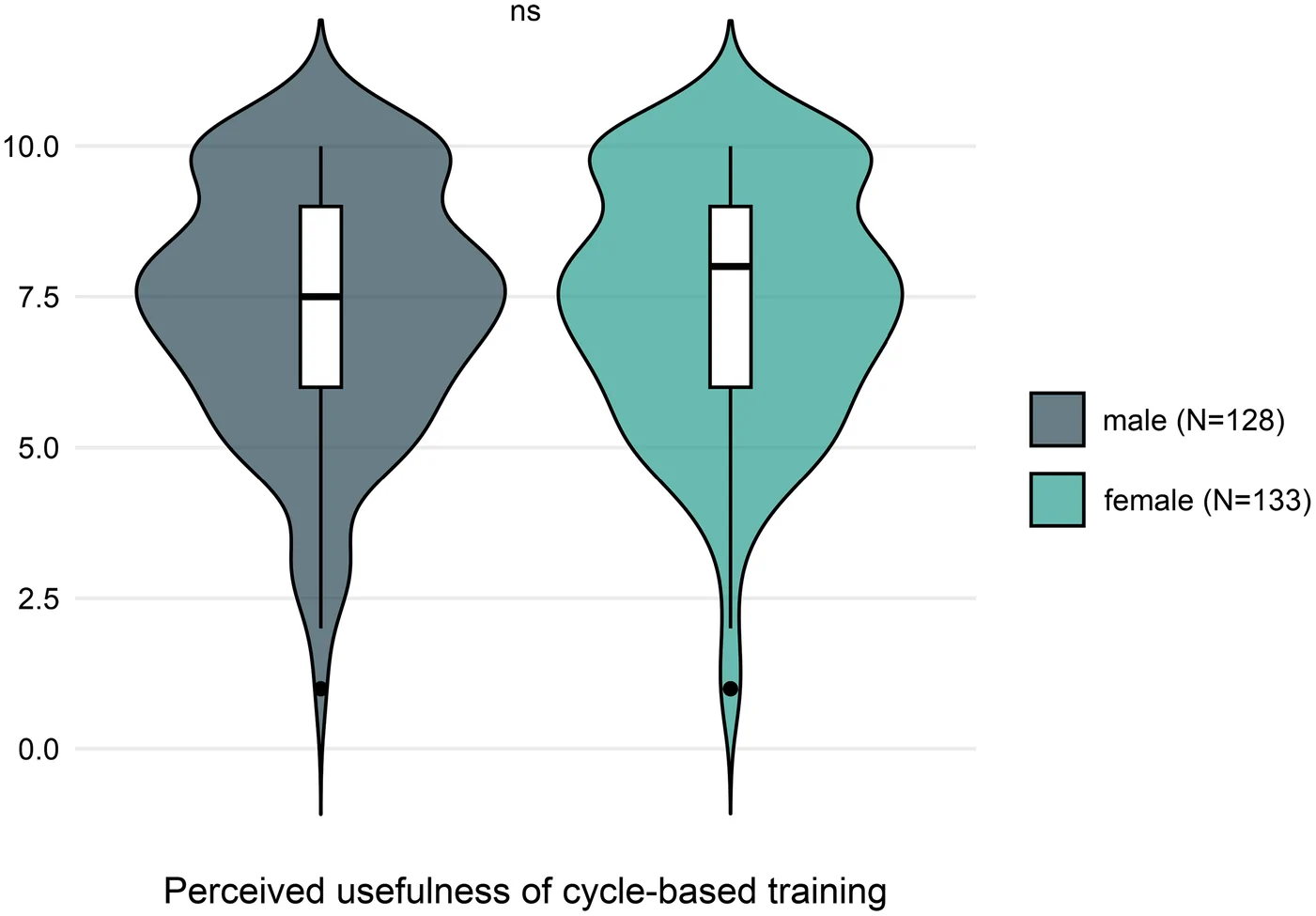
Violin plot comparing the opinions about the usefulness of cycle-based training between male (*N* = 128) and female (*N* = 133) coaches. Male: median 7.5 (IQR 6.0–9.0); female: median 8.0 (IQR 6.0–9.0). No significant difference was detected (Mann–Whitney U, *p* = 0.841; rank-biserial r = 0.01, 95% CI −0.13 to 0.15).

**Figure 4:**
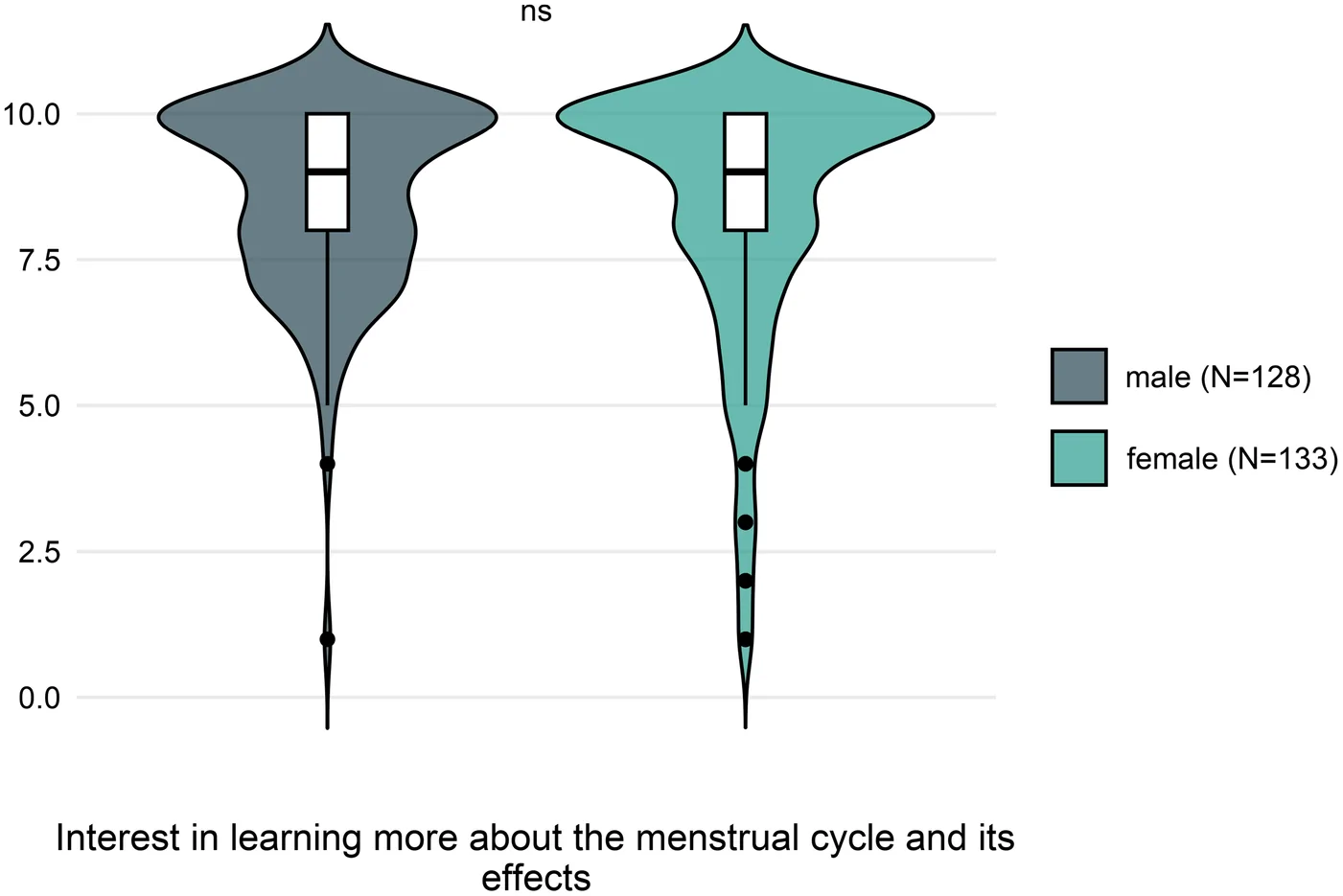
Violin plot comparing interest in learning more about the menstrual cycle and its effects on performance, injury, and recovery (1 = not interested, 10 = very interested) between male (*N* = 128) and female (*N* = 133) coaches. Male: median 9.0 (IQR 8.0–10.0); female: median 9.0 (IQR 8.0–10.0). No significant difference was detected (Mann–Whitney U, *p* = 0.821; rank-biserial r = 0.02, 95% CI −0.12 to 0.15).

Despite knowledge gaps, overall attitudes toward menstrual cycle–based training were strongly positive (Figure 3). Coaches rated the perceived usefulness of cycle-based training at 7.4/10, and willingness to learn more at 8.5/10. Nearly 45% of respondents selected the maximum willingness score (10/10) (Figure 4). Willingness to learn more was high among both male and female coaches, with no significant difference between groups.

### Cycle tracking, tracking tools and implementation of cycle-based

Actual implementation was limited. Forty-five percent of coaches did not track the menstrual cycle at all, and only 2% reported systematic tracking for the entire team. Overall, 10% of coaches actively considered the menstrual cycle in training planning. No statistically significant gender differences were observed in tracking practices, discussion initiation, or inclusion of the menstrual cycle in training planning (Supplementary Figure S1). The most commonly reported barriers to implementation are shown in Table 2; rates were similar between male and female coaches across all categories.

**Table 2:** Barriers for cycle tracking reported by male and female coaches.

Reported Barriers to Implementation of Cycle Tracking		
Barrier	male	female
Lack of knowledge	69.5%	66.9%
Insufficient coverage in coaching education	53.1%	52.6%
Lack of time	37.5%	33.1%
Lack of practical tools	34.4%	36.8%
Perceived stigmatization	20.3%	16.5%

**Table 3:** Self-rated knowledge, perceived usefulness, and willingness to learn, by gender. Values are medians with interquartile ranges and the number of coaches providing a valid response. Effect sizes are rank-biserial correlations with 95% confidence intervals; a positive value indicates higher scores among female coaches.

Item (10-point scale)	Male, median (IQR), n	Female, median (IQR), n	Test	Effect size	p
Self-rated general knowledge of the menstrual cycle	6.0 (5.0–7.0), 128	7.0 (6.0–8.0), 133	Mann–Whitney U	r = 0.44 [0.31, 0.54]	<0.0001
Self-rated knowledge of effects on performance, recovery, injury risk	6.0 (4.0–7.0), 128	6.0 (4.0–8.0), 133	Mann–Whitney U	r = 0.10 [−0.04, 0.23]	0.168
Perceived usefulness of cycle-based training	7.5 (6.0–9.0), 128	8.0 (6.0–9.0), 133	Mann–Whitney U	r = 0.01 [−0.13, 0.15]	0.841
Willingness to learn more	9.0 (8.0–10.0), 128	9.0 (8.0–10.0), 133	Mann–Whitney U	r = 0.02 [−0.12, 0.15]	0.821

**Table 4:** Categorical outcomes by gender, with the number of coaches providing a valid response for each outcome.

Outcome	Category	Male *n* (%)	Female *n* (%)	Valid n	Test	Effect size	p
Athletes use tracking methods	yes	36 (28.1)	32 (24.1)	261	Chi-square	V = 0.05	0.748
	not known	40 (31.2)	45 (33.8)				
	no	52 (40.6)	56 (42.1)				
Systematic recording of the cycle	yes, whole group	1 (0.8)	3 (2.3)	261	Fisher-Freeman-Halton	V = 0.11	0.387
	individually by athletes	58 (45.3)	49 (36.8)				
	not recorded at all	52 (40.6)	65 (48.9)				
	no information	17 (13.3)	16 (12.0)				
Cycle considered in training planning	yes	17 (13.3)	9 (6.8)	261	Chi-square	*φ* = 0.10	0.121
	no	111 (86.7)	124 (93.2)				
Phase-specific injuries observed	yes	13 (10.2)	21 (15.8)	261	Chi-square	φ = 0.07	0.243
	no	115 (89.8)	112 (84.2)				
Specific prevention programmes used	yes	32 (25.0)	14 (10.5)	261	Chi-square	φ = 0.18	0.004
	no	96 (75.0)	119 (89.5)				
Would modify training at elevated risk	+10%	36 (38.3)	41 (38.7)	200	Fisher-Freeman-Halton	V = 0.13	0.741
	1.5x	17 (18.1)	15 (14.2)				
	2x	25 (26.6)	28 (26.4)				
	5x	3 (3.2)	4 (3.8)				
	10x	0 (0.0)	3 (2.8)				
	none	13 (13.8)	15 (14.2)				
Would advise competition withdrawal	+10%	24 (26.1)	11 (10.4)	198	Fisher-Freeman-Halton	V = 0.30	0.002
	1.5x	13 (14.1)	7 (6.6)				
	2x	23 (25.0)	29 (27.4)				
	5x	12 (13.0)	12 (11.3)				
	10x	1 (1.1)	6 (5.7)				
	none	19 (20.7)	41 (38.7)				

### Reporting of cycle-related injuries

Only 34 of 261 coaches (13%) reported clearly observing injuries occurring more frequently in specific menstrual cycle phases. Increased injury susceptibility was most commonly perceived during menstruation, followed by ovulation, with ACL injuries during ovulation mentioned most frequently.

Female coaches were more likely to report phase-specific injury observations, whereas male coaches more often indicated uncertainty or no identifiable pattern.

### Implementation of cycle-based training

When presented with hypothetical injury risk scenarios, 172 of 200 respondents (86%) indicated they would adapt training at some level of increased hypothetical injury risk, distributed as follows: +10% *n* = 77, 1.5 × *n* = 32, 2× *n* = 53, 5× *n* = 7, 10× *n* = 3; 28 respondents would not modify training (Supplementary Figure S2). For competition, 138 of 198 respondents (70%) would consider recommending withdrawal at some threshold: +10% *n* = 35, 1.5× *n* = 20, 2× *n* = 52, 5× *n* = 24, 10× *n* = 7; 60 respondents would not recommend withdrawal.

**Figure 5:**
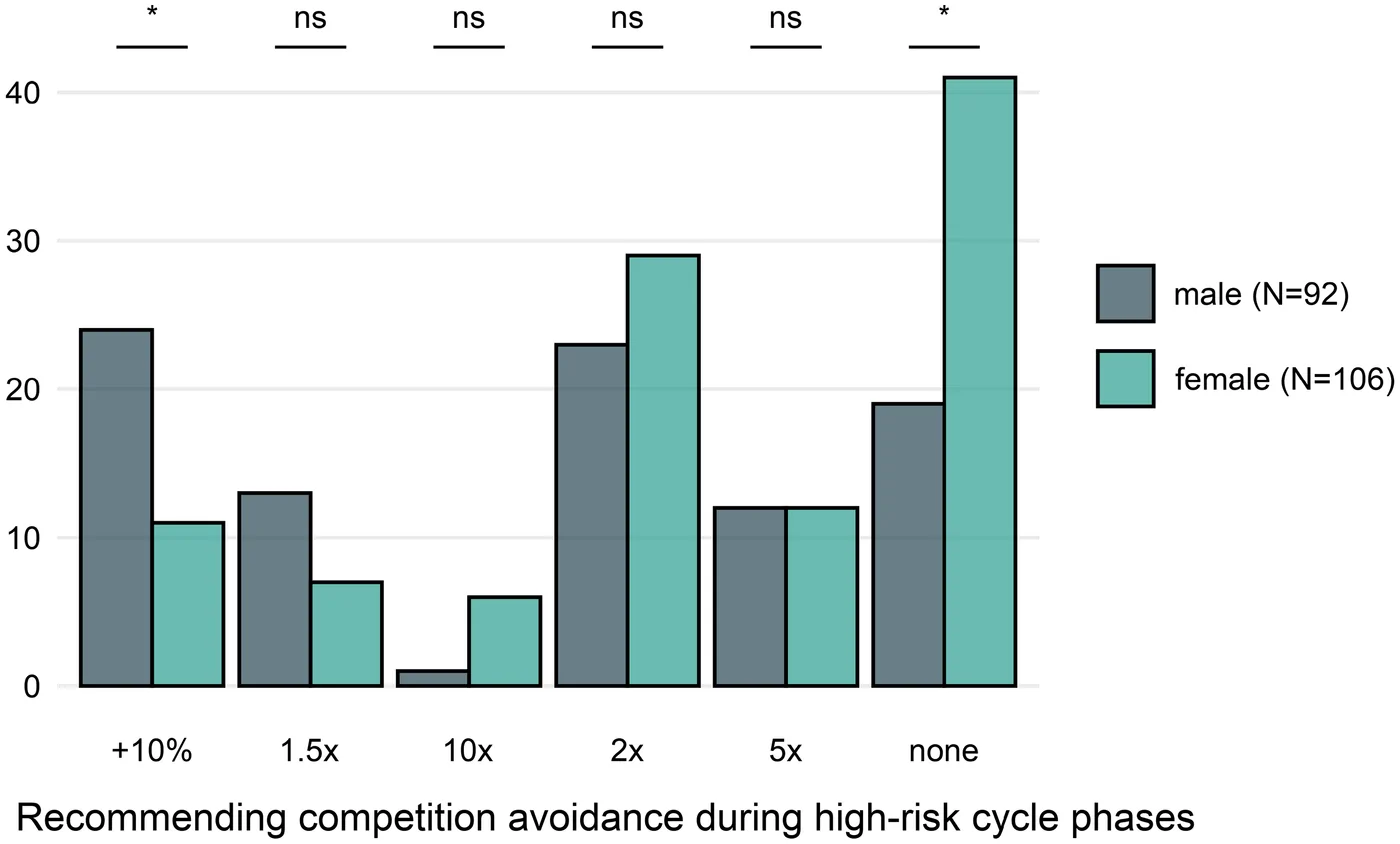
Bar chart comparing recommendations for competition avoidance during high-risk cycle phases between male (*N* = 92) and female (*N* = 106) coaches. Overall difference between groups: Fisher-Freeman-Halton exact test, *p* = 0.002, Cramér's V = 0.30. Significant differences were observed for: +10% (male 26.1%, female 10.4%, adj. *p* = 0.041); none (male 20.7%, female 38.7%, adj. *p* = 0.047). No significant differences for: 1.5× (male 14.1%, female 6.6%, adj. *p* = 0.500); 2× (male 25.0%, female 27.4%, adj. *p* = 1.000); 5× (male 13.0%, female 11.3%, adj. *p* = 1.000); 10× (male 1.1%, female 5.7%, adj. *p* = 0.500). Post-hoc: Chi-square/Fisher's exact tests, Holm-corrected. The response option indicating that withdrawal would be recommended regardless of the level of injury risk was not selected by any respondent and therefore does not appear. Counts by category were: +10% male 24, female 11; 1.5× male 13, female 7; 2× male 23, female 29; 5× male 12, female 12; 10× male 1, female 6; none male 19, female 41.

However, gender-specific differences emerged in competition-related decision-making. Across increasing risk levels, female coaches were more likely to maintain competition participation, whereas male coaches showed a greater tendency to recommend waiving competition at moderate risk increases (Figure 5).

Male and female coaches thus differed in the threshold at which they would recommend competition withdrawal, with female coaches more frequently selecting no withdrawal and male coaches more frequently selecting the lowest threshold.

### Barriers/lack of knowledge and prevention structures

Only a minority of coaches reported the presence of formal guidelines addressing menstrual cycle–related training adaptation or injury prevention within their organization (Supplementary Figure S3). The absence of such guidelines was reported across all coaching levels and sports, indicating that menstrual cycle–specific considerations are not yet institutionally embedded in routine training or competition planning.

Consistent with this finding, the use of preexisting structured injury prevention programs was limited. Only 18% of coaches reported currently implementing established prevention programs (e.g., FIFA 11+, PEP) to reduce knee injuries such as ACL rupture. However, these programs are not adapted to account for menstrual cycle–related risk variations.

**Figure 6:**
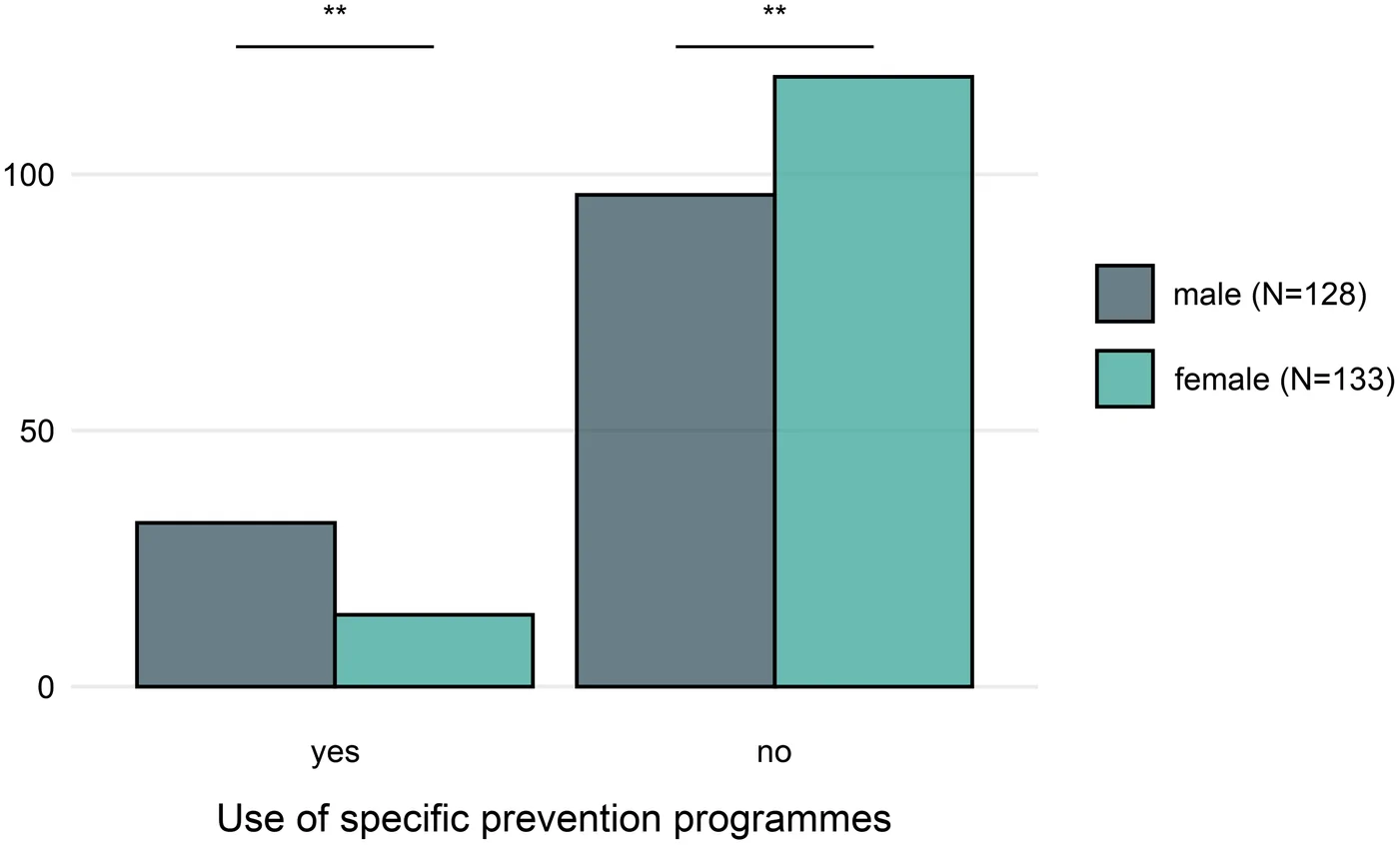
Bar chart comparing the use of specific prevention programs for menstrual cycle–related training adaptation or injury prevention between male (*N* = 128) and female (*N* = 133) coaches. Male respondents were significantly more likely to use specific prevention programs than female respondents [25.0% vs. 10.5%; *χ*^2^(1) = 8.44, *p* = .004, *φ* = 0.18].

Female coaches more frequently reported that no injury prevention programs were available (male 75%, female 90%; *p* = 0.0037), whereas male coaches more often indicated the presence of at least some preventive measures (male 25%, female 11%; *p* = 0.0037); (Figure 6). Nevertheless, no systematic, menstrual cycle–specific prevention strategies were evident in either gender. The most frequently cited reasons for non-use of prevention programs included lack of education, lack of practical guidance, and uncertainty regarding effectiveness, closely mirroring the reported absence of clear guidelines.

Taken together, these findings indicate a substantial gap between theoretical awareness of injury risk and the availability and implementation of structured prevention strategies.

## Discussion

This multi-sport survey, based on nationwide recruitment, provides novel insights into coaches’ perceptions, knowledge, and practices regarding the menstrual cycle and its relationship to training performance and injury risk in female athletes. Female coaches reported higher self-rated general knowledge, whereas self-rated domain-specific knowledge did not differ significantly between genders. Actual implementation, however, did not differ significantly between genders: tracking practices and the inclusion of the menstrual cycle in training planning were comparable, and the use of specific prevention programs was in fact higher among male coaches. Male coaches showed similarly high interest and risk awareness regarding the effect of the cycle on training and reported barriers at rates comparable to those of female coaches.

Across all genders, willingness to participate in targeted education and to modify training and competition decisions was high when supported by clear evidence. This highlights a substantial implementation gap that may be addressed through education and the availability of robust injury risk data to inform training and competition decisions (21).

### Knowledge gaps and gender differences Among coaches

Overall, coaches rated their general knowledge of the menstrual cycle as moderate (mean 6.4/10), while self-rated knowledge related to performance, recovery, and injury risk was lower (mean 5.8/10). This aligns with previous reports indicating that menstrual health remains underrepresented in formal coach education and sports science curricula (22). The consistently higher knowledge scores reported by female coaches likely reflect greater personal exposure to menstrual cycle–related experiences and more frequent informal learning (22).

Importantly, these knowledge differences did not translate into reduced interest among male coaches. On the contrary, male coaches expressed similarly high willingness to learn, suggesting that the observed gaps are not driven by a lack of motivation but rather by insufficient access to education, tools, and evidence-based guidance. Our findings align closely with international data while adding the coaches’ own perspective, which has rarely been surveyed directly. In a Swedish-Norwegian study of 1,086 female athletes, 53% rated their coaches’ knowledge of female athlete health as poor or very poor, and 81% at least partly agreed that female athlete health remains taboo in the athletic community (20). Among Norwegian rhythmic gymnasts, ballerinas, and dancers, 39% rated their coaches’ knowledge as low and only 32% had discussed the menstrual cycle with a coach (23). Notably, a Norwegian intervention study found that coaches rated their own knowledge significantly higher than their athletes rated it, underlining that self-rated knowledge should not be equated with competence — a caveat we apply to our own data (24). In an Indian multi-stakeholder survey, knowledge scores were significantly higher in women than in men, and 92% of athletes reported being comfortable discussing menstrual topics with female coaches compared with 55% with male coaches (25). Our implementation figures lie at the lower end of the international range: in that survey roughly three-quarters of coaches reported integrating the cycle into training planning, whereas in a United Kingdom cycling cohort such discussion was typically confined to a brief note on the training programme rather than systematic tracking (26). The barriers reported by Swiss coaches — knowledge deficits, insufficient coverage in education, lack of tools, and stigma — mirror those identified qualitatively elsewhere, including the male coach–female athlete dynamic and the absence of formal discussion pathways (27, 28). Encouragingly, a tailored educational session for Norwegian junior endurance athletes and their coaches significantly improved both perceived knowledge and perceived ease of communication, indicating that the receptiveness we documented among Swiss coaches can plausibly be converted into competence (24).

### Attitudes versus implementation

Despite generally positive attitudes toward menstrual cycle–based training and high willingness to adapt practices, only a small minority of coaches reported actively considering the menstrual cycle in training planning, and systematic tracking was rare. This discrepancy highlights a clear gap between awareness and implementation. Coaches appear receptive to cycle-informed approaches but lack the confidence, resources, and institutional support needed for implementation (29).

Barriers such as insufficient knowledge, limited coverage in coaching education, lack of time, and absence of practical tools were frequently reported and are consistent with earlier qualitative studies in female athlete health. Perceived stigmatization, although less commonly cited, remains relevant and may further inhibit open discussion, particularly for male coaches working with female athletes (20, 23, 30).

### Perceived injury risk and decision-making

Only a small proportion (male 10%, female 15%) of coaches reported clearly observing phase-specific injury patterns, reflecting the current lack of robust epidemiological data linking menstrual cycle phase to overall injury incidence across sports (1). Nevertheless, menstruation and ovulation were most frequently perceived as higher-risk phases, with ACL injuries during ovulation mentioned most often. These perceptions mirror findings from smaller studies suggesting hormonal influences on ligament laxity, though evidence remains inconsistent and sport-specific (1, 31).

Notably, when presented with hypothetical risk scenarios of not further specified injuries, coaches demonstrated a high willingness to adjust training and, if necessary, to forgo competition. This indicates that in our study, coaches prioritize athlete health over performance when supported by clear risk information. However, gender-specific differences emerged in competition-related decision-making, with male and female coaches differing in the threshold at which they would recommend competition withdrawal. One possible explanation for higher risk-taking in competitions in general is the limited control coaches have over competitions, which are externally scheduled, whereas training can be planned and adapted to the athlete's current condition.

Some research suggests that female coaches may display higher risk-taking in competition contexts (32, 33). This could be influenced by pressures to conform to dominant leadership norms in sport and to establish credibility in a traditionally male-dominated field. At the same time, male coaches working with female athletes may adopt more cautious approaches in certain situations, partly due to heightened awareness of professional boundaries and safeguarding concerns. However, these patterns are context-dependent and should not be generalized across all coaches or settings.

### Lack of structural support and preventive strategies

The near absence of formal guidelines and menstrual cycle–specific prevention strategies across sports and competitive levels represents a major structural limitation. Even established injury prevention programs such as FIFA 11 + and PEP (Prevent injury and Enhance Performance) were infrequently used and rarely adapted to account for potential cycle-related risk variation (1, 34). This finding is particularly relevant given the known effectiveness of structured prevention programs in reducing injury rates, especially for ACL injuries.

The reported lack of guidelines, combined with uncertainty regarding effectiveness, reinforces the need for translational research that bridges physiological findings with practical coaching applications (1, 35). Without clear institutional frameworks, responsibility is placed on individual coaches, further amplifying disparities based on gender, experience, and educational background.

### Practical implications and future directions

Taken together, these findings suggest that while current scientific evidence does not justify universal cycle-based training or competition modifications, there is substantial demand among coaches for clear, evidence-based guidance. Educational interventions integrated into coach certification programs, development of practical communication tools, and provision of simple tracking or decision-support systems may help close the implementation gap.

Several avenues could help to close this gap. A dedicated module on menstrual health might be integrated into coach certification at various levels, covering cycle physiology, the current state and the limits of the performance evidence, and practical communication skills. Given the asymmetry reported elsewhere in athletes’ comfort when discussing menstrual health with male compared with female coaches, such provision could usefully address confidence and framing for male coaches specifically, rather than assume that general instruction is sufficient. Federations might also consider offering a short standard tracking template together with guidance on data protection, an aspect that respondents themselves raised as a barrier. Where cycle-phase information is collected, integrating it into existing injury surveillance would seem more practicable than establishing a parallel system. It would be important that any such guidance make clear that current evidence does not support mandatory cycle-based modification of training or competition schedules. National federations and Swiss Olympic are well placed to take these steps forward, perhaps beginning with a shared position statement and an educational offering, followed in time by tracking templates and cycle-phase fields in existing injury registries, and, in the longer term, by prospective sport-specific injury surveillance incorporating menstrual cycle and hormonal contraceptive data. In the absence of such structures, implementation is likely to remain dependent on individual initiative, which may perpetuate the variation we observed across coaches of differing gender, experience, and educational background.

Future research should prioritize large-scale, sport-specific injury surveillance studies that incorporate menstrual cycle and hormonal data, as well as intervention studies evaluating individualized, symptom-based approaches. Such evidence is essential to move beyond perception-driven decision-making toward structured, athlete-centered strategies.

## Strengths and limitations

Strengths of this study include its nationwide scope, the inclusion of multiple sports and competitive levels, and balanced representation of male and female coaches. Limitations include reliance on self-reported data, and the indirect dissemination of the questionnaire through the sport federations. Because the invitation was forwarded by the federations, the total number of coaches reached is unknown; consequently, a response proportion could not be calculated and the characteristics of non-responders could not be assessed. This may have introduced selection bias, as coaches with a pre-existing interest in menstrual health may have been more likely to participate. Further limitations include imbalanced representation of different sports, and the cross-sectional design, which precludes causal inference. Recruitment was nationwide in approach, but the resulting sample cannot be assumed to be nationally representative: the number of federations that forwarded the invitation and the number of coaches who received it are unknown. The geographic and linguistic distribution of respondents was not recorded, and given the differing sporting structures across Switzerland's language regions, a regional imbalance cannot be excluded. Knowledge was assessed by self-rating only; no objective knowledge test was administered, and we therefore cannot determine whether the observed differences reflect actual knowledge, personal experience, or differing confidence in self-assessment. The questionnaire was purpose-built and not formally validated; internal consistency, test-retest reliability, and construct validity were not assessed. Finally, the hypothetical risk scenarios specified relative increases in injury risk without a baseline absolute risk, injury type, severity, time frame, or sporting context; a twofold increase carries very different practical meaning depending on the baseline, and responses should therefore be read as indicating a willingness to act in principle rather than as a calibrated measure of risk tolerance.

## Conclusion

Among the Swiss coaches who responded to this survey, male and female alike, there was broad recognition of the potential relevance of the menstrual cycle to athletic performance and injury risk, together with a strong willingness to adapt training practices when supported by evidence. However, limited self-rated knowledge, insufficient formal education, and the absence of institutional guidelines currently seem to hinder effective implementation. Addressing these gaps through targeted education and the development of robust injury-risk data might represent a critical opportunity to enhance the health, well-being, and performance of female athletes.

## Data Availability

The datasets presented in this article are not readily available because the deidentified data and data dictionary are available from the corresponding author upon reasonable request and are not deposited in a public repository. Requests to access the datasets should be directed to christian.tinner@insel.ch.
