# Supplementary material for: Importance of respect for the menstrual cycle in sports training and competition: a nationwide multisport coach questionnaire
Source: Front Sports Act Living. 2026 Sep 3;8:1906962. doi: 10.3389/fspor.2026.1906962 (PMC13582195; doi:10.3389/fspor.2026.1906962)
Supplement: Supplementary file 1 [file Datasheet1.pdf]

Dimension	Question Number	Question	Response Option/Scaling	Response requirement
**Demographic Information (1)**	1	Gender	Man; Woman; Non-binary	Required
	2	How old are you?	<18; 18-24; 25-40; 41-64; 64+	Required
	3	In what capacity are you currently working as a trainer?	Full-time; Part-time; Volunteer	Required
	4	In which sport do you work as a coach?	Open text field	Required
	5	At what level(s) do you work as a trainer?	National; Regional; Club; Self-empolyed (Multiple responses allowed)	Required
	6	Number of weekly training sessions for your female athletes?	1-3; 4-6; 7-10; 10+	Required
	7	How many hours per week do you spend working as a trainer?	1-5; 6-10; 11-20; 21-30; Full-time	Required
**Knowledge and mindset (2)**	8	How extensive would you say your knowledge of the menstrual cycle is? (1=very little, 10=very extensive)	10-point Likert scale	Required
	9	Are you aware of the effects of the menstrual cycle on performance/recovery/risk of injury? (1=no knowledge, 10=extensive knowledge)	10-point Likert scale	Required

	10	Where do you get information on this topic?	Information material from the association; Training and continuing education; Studies; Magazines; Social Media; I have not yed obtained any information on this subject.; Other (Multiple responses allowed)	Required
	11	Do you find cycle-dependent training reasonable? (1=not useful, 10=very useful)	10-point Likert scale	Required
	12	Would you be willing to learn more about the menstrual cycle and performance, injury, and recovery? (1=not willing, 10=very willing)	10-point Likert scale	Required
**Tracking and Tools (3)**	13	Do your athletes use tracking methods?	Yes; No; No information/knowledge	Required
	14	What tools are used to track the menstrual cycle?	Apps; Wearables (e.g. smart ring or trackle); Calendar; No information; Other (Multiple responses allowed)	If 13 = Yes Required
**Implementation (4)**	15	Who is bringing up the topic of the menstrual cycle?	Athletes; Coaches; Club; Association; No one; Other (Multiple responses allowed)	Required

	16	Is the menstrual cycle systematically recorded?	Yes, for the entire training group; No, the athletes do this individually; No, the menstrual cycle is not recorded at all; No information	Required
	17	Is the menstrual cycle taken into account when planning training?	Yes; No	Required
	18	For what reasons do you take the menstrual cycle into account when planning training?	Performance; Recovery; Risk of injury; Wellbeing; Other (Multiple responses allowed)	If 17 = Yes Required
	19	Are there any guidelines that are followed in this regard? (e.g., from Swiss Olympics)	Yes; No	If 17 = Yes Required
	20	What are these guidelines?	Open text field	If 19 = Yes Required
	21	Are there specific training adjustments for menstruation, the follicular phase, ovulation, and the luteal phas	Yes; No, it is tailored to the individual athlete.	If 17= Yes Required
	22	Describe the training adjustments for the 4 phases: menstruation, follicular phase, ovulation, luteal phase.	Open text field	Optional

**Menstrual cycle and injury (5)**	23	Do you observe differences in susceptibility to injury during the different phases of the menstrual cycle?	Increased risk during menstruation; Increased risk during the follicular phase; Increased risk during ovulation; Increased risk during the luteal phase; No; Other (Multiple responses allowed)	Required
	24	Are there injuries that occur more frequently during certain phases (e.g., cruciate ligament injuries during ovulation)?	Yes; No	Required
	25	Describe the type of injury and the corresponding phase of the cycle.	Open text field	If 24 = Yes Optional
	26	How man injuries in total per seaseon are there in your training group/team	Open text field	Required
	27	How many athletes are in the training group/team?	Open text field	Required
	28	Are specific prevention programs implemented? (e.g., FIFA11+, PEP, Sportsmetrics)	Yes; No	Required
	29	Which prevention program is used?	Open text field	If 28 = Yes Optional

	30	Would you adjust your training if, during a phase of your cycle, you had a	10% higher risk of injury; 1.5 times greater risk of injury; twice the risk of injury; 5 times greater risk of injury; 10 times greater risk of injury; I wouldn't adjust it	Optional
	31	Would you recommend refraining from competition if, during a phase of the cycle, there is a	10% higher risk of injury; 1.5 times greater risk of injury; twice the risk of injury; 5 times greater risk of injury; 10 times greater risk of injury; Regardless of the risk of injury; I would not recommend withdrawing from a competition.	Optional
**Barriers (6)**	32	In your opinion, what hinders the implementation of cycle-controlled training?	Lack of knowledge; Insufficient discussion in education and training; Lack of time; Lack of tools; Dataprotection; Stigmatization; Lack of interest; Other (Multiple responses allowed)	Required
**Additional (7)**	33	Do you have any further thoughts or opinions you would like to share on this topic?	Open text field	Optional
